# Acoustic-prosodic measures discriminate the emotions of Brazilian portuguese speakers

**DOI:** 10.1590/2317-1782/e20240116en

**Published:** 2025-08-04

**Authors:** Alexandra Christine de Aguiar, Ana Carolina Constantini, Ronei Marcos de Moraes, Anna Alice Almeida

**Affiliations:** 1 Programa de Pós-graduação em Modelos de Decisão e Saúde, Universidade Federal da Paraíba – UFPB - João Pessoa (PB), Brasil.; 2 Departamento de Desenvolvimento Humano e Reabilitação, Universidade Estadual de Campinas – UNICAMP - Campinas (SP), Brasil.; 3 Departamento de Estatística, Universidade Federal da Paraíba – UFPB - João Pessoa (PB), Brasil.; 4 Departamento de Fonoaudiologia, Universidade Federal da Paraíba – UFPB - João Pessoa (PB), Brasil.

**Keywords:** Voice, Emotion, Speech Acoustics, Prosody, Emotion Recognition in Voice

## Abstract

**Purpose:**

To verify if there is a difference in acoustic-prosodic measures in different emotional states of speakers of Brazilian Portuguese (BP).

**Methods:**

The data sample consisted of 182 audio signals produced by actors (professionals or students), from the semi-spontaneous speech task “Look at the blue plane” in the various emotions (joy, sadness, fear, anger, surprise, disgust) and neutral emission. Values were extracted from acoustic-prosodic measures of duration, fundamental frequency and intensity of the various emotions. The Friedman comparison test was used to verify whether these measures are able to discriminate emotions.

**Results:**

The prosodic-acoustic analysis revealed significant variations between emotions. The disgust emotion stood out for having the highest rate of utterance, with higher values of duration. In contrast, the joy exhibited a more accelerated speech, with lower values of duration and greater intensity. Sadness and fear were marked by lower intensity and lower frequencies, and fear presented the lowest positive asymmetry values of z-score and z-smoothed, with less elongation of the segments. Anger was highlighted by the higher vocal intensity, while surprise recorded the highest values of fundamental frequency.

**Conclusion:**

The acoustic-prosodic measures proved to be effective tools for differentiating emotions in CP speakers. These parameters have great potential to discern different emotional states, broaden knowledge about vocal expressiveness and open possibilities for emotion recognition technologies with applications in artificial intelligence and mental health.

## INTRODUCTION

The interface between voice and language is related to the point of intersection between vocal production and linguistic expression. It involves the connection between the ability to produce vocal sounds and the ability to use these sounds in a structured and meaningful way, following specific linguistic rules^(1)^. The voice is used to convey language, that is, to express words and meanings according to the rules and linguistic conventions. It can differentiate the emotional state expressing personality traits, feelings, physical and mental health status, among others^(2-4)^.

Language influences the way we use our voice. The grammatical structure, vocabulary and linguistic patterns determine how we organize and express our ideas from the voice^(5)^. Therefore, voice and language are intrinsically related, and their interaction is critical for effective communication and emotional expression.

Several studies have contributed to the fact that some authors proposed that six basic emotions (happiness/joy, fear, anger, sadness, disgust/disgust, surprise) should be universally recognized in the face by human beings, for presenting specific configurations, expressed in a similar way in different cultures^(6,7)^. These emotions when combined generate a spectrum of emotional states.

Each emotional state causes changes in the vocal tract and momentarily alter the physiology of voice production, which interferes with breathing control, vertical positioning of the larynx, in the relative relaxation of vocal folds and in the positioning and relaxation of pharyngeal and tongue muscles, which may result in voice modification^(8)^. These variations of the human voice, when an individual experiences a certain emotional state, can involve both aspects of vocal quality and related attributes of prosody^(9)^. This can be defined as a set of speech properties, which is beyond the segment level, and is usually studied from the analysis of three classical phonetic-acoustic parameters: duration, fundamental frequency (f_o_) and intensity^(9-11)^. For some authors, prosody results from the coupling of syntactic, semantic and discursive information and the constraints of a speech production system^(12,13)^. The prosodic variations transmit information to the significance of expressiveness and mark the characteristics of a vocal dynamic, but little is known about this information in emotions for Brazilian Portuguese (BP).

Some voice banks were developed with the variation of emotions in different languages/cultures, such as the Berlin Database of Emotional Speech (EMO-DB)^(14)^_,_ Interactive Emotional Dyadic Motion Capture (IEMOCAP)^(15)^_,_ Sustained Emotionally colored Machine-human Interaction using Nonverbal Expression (SEMAINE)^(16)^ and Remote Collaborative and Affective interactions (RECOLA)^(17)^_._ The main characteristics that stand out in the differentiation of emotions in these data sets are the acoustic and prosodic characteristics, such as pitch, energy and duration. In addition, cepstral coefficients such as the Mel-Frequency Cepstral Coefficients (MFCC) are important measures to optimize emotional identification^(18)^.

Recently, a voice bank in the various emotions was developed from native speakers of BP, the EMOVOX-BR^(19)^. There was the validation of this bank through the perceptive-auditory judgment of expert judges. This study indicated that acoustic aspects, such as pitch and loudness variation, were essential for the differentiation of emotions, in addition to their valence and power^(19)^.

The analysis of acoustic-prosodic aspects of emotions is an area of growing interest in speech and communication sciences, offering new perspectives on how emotions are expressed and perceived through voice^(20-22)^, and therefore the present study seeks to collaborate with the identification of vocal parameters and speech specific to each emotional state, as well as recognize the voice as a biological signal rich in information that can be instrumental in detecting emotional patterns.

The investigation of emotional prosody involves both how acoustic modulations are produced and how they are perceived, providing a broader understanding of the relationship between linguistic and emotional processes in human communication^(23)^, going beyond the semantic content of words. Understanding these elements is essential to unravel the complexity of human communication, in which the emotional dimension significantly influences social interaction. In addition to its theoretical impact, the study of emotional prosody presents practical applications in advanced technologies for emotion recognition, artificial intelligence, and clinical diagnoses of communication disorders and mental health^(21,22)^.

These findings have the potential to boost the development of human-machine interaction systems and automatic voice emotion detection, since prosody involves elements that carry important information about the emotional state of the speaker^(23)^. The expression of emotions affects these parameters in a distinct way, allowing machine learning models to use these variations to identify and classify emotions accurately^(24)^. This ability is essential to create systems capable of adjusting their responses according to the emotional state of the user, promoting more human and efficient interactions.

These advances have fundamental applications for a wide range of industries such as call centers, voice recognition apps, web movies and mobile communication. It is believed that the analysis of prosodic-acoustic parameters could reveal phonic differences between the emotional variations of BP, contributing to the development of systems of synthesis and speech recognition more adapted to the local language and culture.

Given the above, questions arise: Is it possible to discriminate emotions from acoustic-prosodic measures in BP speakers? What prosodic aspects characterize the different emotional states? Are there differences in duration, frequency and intensity between emotions? Thus, the objective of this study was to verify if there is difference in acoustic-prosodic measures in different emotional states of BP speakers.

## METHODS

This is an observational and cross-sectional research, evaluated and approved by the Research Ethics Committee of the Health Sciences Center of a higher education institution in Brazil, under number 3.304.419/2018.

The data set for analysis was composed of 182 sound signals, produced by 26 professional actors and Brazilian students, of both sexes, with an average age of 27 years, living in the states of Paraíba, São Paulo, Rio Grande do Sul, Ceará, Roraima, Mato Grosso and the Federal District, which belong to the Brazilian Voice Bank in the Variations of Emotions - EMOVOX-BR^(19)^.

For the construction of EMOVOX-BR^(19)^ voice samples were recorded from native speakers of BP expressing different emotions, such as joy, sadness, fear, anger, surprise, disgust and neutral emission. Participants received detailed instructions on the recording procedure and then performed voice collection. Three different speech tasks were recorded: 1) extended vowel emission /ε/, 2) automatic counting from 1 to 10 and 3) semi-spontaneous speech of the phrase “Look at the blue plane.”, which is part of the Consensus Auditory Perceptual Evaluation of Voice - CAPE-V^(25)^. Each participant performed these tasks in the six basic emotions. Which generated a total of 1,638 sound signals.

For validation, we chose to use the phrase “Look at the blue plane.” collected through smartphone. All audios were submitted to the signal-to-noise ratio (SNR) analysis and obtained values higher than the reference standard, that is, the value of SNR equal or greater than 30dB. We selected 182 audio signals for the stage of perceptive-auditory judgment. These vocal samples were evaluated by speech-language judges, who obtained high levels of precision in the identification of the six basic emotions.

This study analyzed the acoustic-prosodic measures of the same 182 audios used in the validation of EMOVOX-BR, which was performed from the perceptive-auditory judgment by expert judges. While the validation study of EMOVOX-BR emphasized the precision of human perception to identify emotions, this work deepens in the quantitative analysis of emotional prosody, mainly in the behavior of acoustic parameters of duration, f_o_ and intensity. These parameters are considered the most robust elements for speaker discrimination and offer a technical and measurable perspective of the emotional aspects of voice^(10,13)^.

The duration of an emotion can vary from short moments to long periods, depending on the intensity of the emotional stimulus, the person’s ability to regulate emotions and the context in which the emotion occurs^(20)^. Duration is essential to identify variations in the speech cadence, enunciation structure and temporal organization of phrases^(26)^.

For duration analysis, all speech samples were manually segmented into Vowel-Vowel (unit VV) units, syllable-sized units that comprise a segment from the beginning of a vowel to the beginning of the immediately following vowel, including the consonants between them^(27)^.

The speech task used was segmented into four parts, being: [Al] [auav] [iaNU] [az]. To extract this measure, considering the calculation of the normalized duration of VV units, we used the script SG Detector^(28)^. The script presents a reference table with averages and standard deviations of the phonic segments for the BP to calculate the duration value, z-score and z-score smoothed units VV throughout the statement, which generates a segmentation of the phrasal groups of statements. Segmentation is done by calculating the standard deviation of the duration averages of VV units, which are normalized by the z-score calculation.

The z-score value indicates the number of standard deviations from the average of an information point that in this case would be the variations of the BP, that is, it is a proportion of the number of standard deviations below or above the BP, which means a gross score. A z-score is called standard score and may well be placed on a common scatter curve and extends from - 3 to + 3 deviations^(29)^.

The z-score values softened allow to attenuate local variations of duration due to the fall of duration in units VV post-tonic and/or duration of headphones very different from the durations ratio of the BP^(27)^. This value corresponds to the smoothed of five points applied to the z-score data sequence, which allows observing more precisely the duration prominences. The aim is to verify how much the duration values obtained in the corpus of EMOVOX-BR^(19)^ varied according to the intrinsic durations table for the phones of the BP.

When considering the number of speech segment units VV divided by the total sum of their duration, we obtain the rate of elocution^(30)^. It influences the perception of speech rhythm, with slower rates associated with syllabation, elongation of final sounds and pauses, while faster rates tend to reduce these phenomena^(28)^. The relationship between the duration of the emission and the rate of utterance was explored to observe the variation in speech speed according to different emotions.

The f_o_, measured in Hertz (Hz), is defined by the number of vibrations produced by vocal folds per second and is directly related to pitch perception^(31)^. This parameter allows a detailed analysis of the intonation and tonal variation along utterances, providing information about vocal control and quality^(32)^. For the analysis of f_o_ were measured the mean, maximum, minimum, standard deviation (f_o_ sd) and variation (f_o_ range) in each statement.

Intensity, measured in decibels (dB), is associated with the strength or degree of emotional activation experienced by the speaker and is related to the vocal energy employed during speech^(31)^. Its measurement helps to understand the energy of the emission and the modulation of force throughout the speech, contributing to studies of prosody and vocal expressiveness^(13)^. For the intensity analysis, the mean, minimum and maximum values were extracted for each signal and, subsequently, the data were compared to each emotion. The f_o_ and intensity measurements were extracted with the help of the VoxMore plug-in^(33)^.

This study investigates whether the acoustic-prosodic measures of duration, f_o_ and intensity vary significantly between different emotions such as joy, sadness, fear, anger, surprise, disgust and neutral emission in BP speakers. Based on the literature, which demonstrates that emotions influence acoustic parameters in a distinct way^(20-22)^, the objective is to test these variations using a set of data from native speakers.

The PRAAT program, version 5.4.04, was used for data extraction. The Friedman test with post hoc of Dunn was selected to verify whether these differences in acoustic parameters between emotions were statistically significant, contributing to a better understanding of the relationship between the acoustic-prosodic aspects and emotional expression, after the results extracted and identified the groups that differed, a descriptive analysis of the variables was performed to identify which prosodic parameter and emotion that stood out in the group. All analyses were performed using the software Statistical Package for Social Sciences (SPSS), version 24, and the significance level of 5% was used.

## RESULTS

The prosody analysis included data extracted according to segmented VV units, the contrast of the variations of f_o_ and intensity after analysis.

There was a significant difference in the duration of segments according to emotion (Table 1). In relation to the total duration of the emission, the highest value was found in the disgust emotion (340.63 ms), which presented the highest rate of elocution. On the other hand, joy emotion had the lowest duration value (264.73 ms), corresponding to the lowest rate of elocution, which indicates a direct relationship between the total time of emission and the speed of speech in the different emotions.

**Table 1:** Comparison of duration parameters for each VV unit of the statement “Look at the blue plane”, produced in different emotions

Duration (ms)	Emotion							p-value
	Joy	Fear	Sadness	Anger	Surprise	Disgust	Neuter	
Mean	264.73	266.81	284.83	270.32	276.7	340.63	274.83	0.024*
Al	172.92	156.08	170.73	175.04	174.81	220.88	168.5	0.001*
Auav	437.73	481.81	528.46	466.27	469.04	605.15	495.69	0.066*
iaNU	212.46	204.27	215.92	205.23	198.88	239.92	206.77	0.317
az	238.81	225.08	224.19	234.73	264.08	296.58	228.35	0.000*

Caption: Friedman test; *Significant values (p < 0.05); ms = milliseconds

The longest duration in all four parts of the VV units was again found in the disgust emotion when analyzing the duration of each segment individually (Al - 220.88 ms; auav - 605.15 ms; iaNU - 239.92 ms; az - 296.58 ms). The shorter duration per segment significantly differentiated all emotions from each other. The shorter duration of the first segment was observed in fear emotion (Al - 156.08 ms), that of the second segment was shown in joy (auav - 437.71 ms), that of the third in surprise (iaNU - 198.88 ms) and that of the last segment in sadness (az - 224.19 ms). There was no significant difference in the third segment [iaNU] in the varied emotions in all parameters related to duration (Table 1).

There was a significant difference when comparing the z-score and z-smoothed values of the first, second and last segment of the z-score in the various emotions (Table 2).

**Table 2:** Comparison of z-score and smoothed z-score parameters for each VV unit of the statement “Look at the blue plane”, produced in the various emotions

		Emotion							P-value
		Joy	Fear	Sadness	Anger	Surprise	Disgust	Neuter	
Z-score	Mean	0.95	0.74	1.23	0.98	1.10	2.74	0.99	0.014*
	Al	3.37	2.52	3.26	3.48	3.47	5.79	3.15	0.024*
	auav	0.22	0.85	1.54	0.62	0.98	2.67	1.06	0.079*
	iaNU	-0.63	-0.99	-0.48	-0.95	-1.22	0.56	-0.88	0.317
	az	0.83	0.55	0.56	0.75	1.19	1.95	0.63	0.001*
Smoothed Z-score	Mean	0.94	0.78	1.28	1.00	1.13	2.8	1.03	0.015*
	Al	2.32	1.97	2.69	2.53	2.64	4.75	2.45	0.002*
	auav	1.27	1.41	2.11	1.57	1.81	3.71	1.75	0.028*
	iaNU	-0.17	-0.31	0.13	-0.29	-0.3	1.26	-0.19	0.174
	az	0.34	0.05	0.20	0.19	0.39	1.49	0.13	0.055*

Caption: Friedman test; Significant values (p < 0.05)

Table 2 presents the disgust emotion with the highest positive asymmetry values of z-score and z-smoothed. It was found that fear emotion has the lowest positive asymmetry values among all emotions. It is possible to observe negative values of z-score and z-smoothed in the third segment [iaNU] in the emotions joy, fear, sadness, surprise, anger and neutral emission. Already in the disgust emotion there was an elongation of the segments compared to the reference values (Table 2).

There was significance in the comparison of all acoustic measures related to voice frequency in the various emotions analyzed. It was found that the highest average f_o_ was in surprise emotion (284.43 Hz), followed by joy (268.54 Hz), and the lowest in sadness (160.91 Hz) and neutral (163.44 Hz). The highest maximum frequency was presented in surprise emotion (360.36 Hz) and the lowest minimum frequency was marked by sadness (77.91 Hz). The fear emotion had the highest f_o_ range (178.11 Hz) and the lowest in the sadness emotion (116.73 Hz) (Table 3).

**Table 3:** Comparison of f_o_ values ​​for the various emotions (average, minimum, maximum and range)

f_o_ (Hz)	Emotion							P-value
	Joy	Fear	Sadness	Anger	Surprise	Disgust	Neuter	
Mean	268.54	228.29	160.91	213.23	284.43	188.47	163.44	0.000*
min	166.42	102.05	77.91	137.10	183.61	135.15	80.91	0.000*
max	344.10	281.86	203.80	287.79	360.36	269.94	194.65	0.000*
range	174.67	178.11	116.73	145.79	176.75	134.79	122.88	0.000*

Caption: Friedman test; *Significant values (p < 0.05); fo = fundamental frequency; Hz = hertz

There were comparisons of the independent samples of mean, minimum and maximum intensity significant in the various emotions (Table 4). The highest peak intensity was in anger (82.35 dB), followed by joy (66.79 dB), and the lowest in fear (35.5 dB) and sadness (36.78 dB). There was a statistically significant difference in the comparison of the average intensity, with the highest recorded intensity in the emotion of joy (66.79 dB) and the lowest in the emotion of sadness (60.23 dB).

**Table 4:** Comparison of intensity values ​​for the various emotions (average, minimum and maximum).

Intensity	Emotion							P-value
	Joy	Fear	Sadness	Anger	Surprise	Disgust	Neuter	
Mean	66.79	63.8	60.23	65.58	65.08	61.91	61.28	0.001*
min	55.29	35.5	36.78	54.87	47.86	43.12	41.89	0.001*
max	80.11	79.64	77.02	82.35	79.72	77.77	79.8	0.001*

Caption: Friedman test; *Significant values (p < 0.05); dB = decibel

Chart 1 shows a synthesis of the prosodic variations present in the various emotions, within the context of BP speakers, that is, the characteristics that most mark each emotion and differentiate them from the others.

**Chart 1:** Prosodic variations in different emotions in Brazilian Portuguese

Emotions	Acoustic-prosodic measures		
	Duration	Frequency	Intensity
Joy	↓	↑	↑
Anger	ND	ND	↑
Surprise	ND	↑	ND
Sadness	ND	↓	↓
Fear	↓	ND	↓
Disgust	↑	ND	ND
Neuter	ND	↓	ND

Caption: ↑ = higher; ↓ = less; ND= not discriminated

## DISCUSSION

The characteristics of the acoustic-prosodic parameters present variations that reflect on the expression of emotions. The knowledge of these measures contributes to the construction of the definition of common emotional variations in vocal signals, can assist in clinical diagnostic aspects and favor the creation of models for recognition of emotions from the voice^(34)^.

Several banks of voices that incorporate emotional variation were developed, covering populations of actors in different languages and cultures^(14-17)^. However, these banks of voices usually focus only on the analysis of traditional acoustic variations and little is known about other types of measures, such as: deepening in the acoustic-prosodic and perceptual on the part of speech therapists and in the impact of judgment by hearing experts, as well as the possibility of finding recognition of voice patterns common to each of the emotions.

The inclusion of prosodic measures in the analysis of emotional voice variation in a data set validated by expert judges is fundamental for a comprehensive and accurate analysis of human emotional expression^(35)^. This increases the validity of data and improves the ability to generalize to different contexts and populations, as well as improving the effectiveness and robustness of automatic emotion recognition models. Thus, it can contribute to a more holistic understanding of human emotional processes and how they are expressed and perceived through speech^(36)^.

Prosody has its dimension based on the suprasegmental aspects of speech, which relate to variations in duration, frequency and intensity^(10,37)^. The present study sought to explore whether there are differences in prosodic variations in different emotions from the voice, the data reported in this study confirmed that these parameters are important factors to define emotions through the objective analysis of vocal signals. Of the nineteen parameters that presented significant results, twelve concern the rhythmic structure of the varieties studied, four refer to f_o_ and three to intensity, which shows that the three classical parameters of prosody study present distinctions between the various emotions.

There was a difference between the duration measures in the speech task in the various emotions. The disgust presented higher values in the average duration, as well as in all segmentations of VV units, that is, it was the emotion with the highest rate of speech elocution. As for the shorter average duration, the joy emotion presented lower values, so it is the emotion with more accelerated speech speed. Changes in speech speed alter the phonetic characteristics of the signal, thus becoming a parameter capable of differentiating emotions^(38)^.

When analyzing the parameter of shorter duration per segment, it was observed that each segment presented shorter duration in different emotions, varied between fear, joy, surprise and sadness. Speakers of a language do not always express emotions in the same way, with the same levels of activation^(39)^. Each emotional state can be defined as a linear combination of some axes, such as activation (or excitation) and power (or power). Activation measures the individual’s degree of excitement in expressing emotion. Potency refers to the strength of emotion^(40)^. Then, each of the basic emotions can generate different levels of activation and power in the speaker, depending on its manifestation can cause differentiation in speech speed.

For the data obtained from z-score and z-smoothed, significant differences were observed in relation to the speech variations of BP between emotions. The disgust emotion presented higher values of positive asymmetry. This fact indicates that speakers increase the duration of some VV units during the emission of this emotion. It means that in the disgust there were more elongations in the duration of the units, which results in the higher positive value asymmetry, and thus relates to a more slowed speech^(27)^. Fear was the emotion with lower values of positive asymmetry among all emotions, that is, there are less elongations in the duration of units.

As for the negative values of z-score and z-smoothed found in the third segment [iaNU] in the emotions joy, fear, sadness, anger, surprise and neutral emission. These values below the reference standards mean that there was a shortening in the duration of the segments for these emotions^(27)^. The acceleration of the segment [iaNU] was a strategy used in the emission of emotions, so it did not present a significant difference in the comparison. In the case of emotion disgust, there was an elongation of the segments compared to the reference values, thus confirming the results found for the elocution rate.

Studies developed in different languages reveal that the faster utterance is associated with emotions of greater excitement. On the other hand, a slower speech is usually linked to states of reflection or calm. In tonal languages, such as Mandarin, the rate of utterance not only affects prosody, but can also alter the meaning of words, being crucial for the correct interpretation of the speaker’s intentions^(41)^. Cultural and regional variations play an important role in the modulation of speech speed, besides indicating emotions, the rate of elocution may reflect specific cultural and linguistic norms^(41-43)^. Therefore, duration analysis offers a deeper understanding of emotional and communicative patterns, varying according to language and cultural context.

It is found that the average and maximum values of f_o_ allow to distinguish the surprise emotion and joy, which positions them between the upper bands of f_o_. These emotions are characterized by a greater tonal variation, typical of emotional states of higher excitement or positive^(44)^, as opposed to sadness and neutral, which have low minimum and average values of f_o_. Joy is a positive valence emotion and surprise is a bivalent emotion. The f_o_ characteristics discriminate emotions in the valence dimension with greater precision^(45)^. For EMOVOX-BR^(19)^, the expert judges indicated that the surprise of this bank was a positive valence emotion. This fact justifies, therefore, the emotions of positive valence have as characteristic variation of pitch in ascending curve. The f_o_ is directly related to laryngeal function, therefore, changes in intonation and airflow produced by the vocal tract due to emotional state can be identified in the acoustic-prosodic analysis of the vocal signal^(46)^. The variation of f_o_ is important in fear, surprise and joy, because they move away from sadness, disgust and neutral.

The variation in pitch along the speech was essential to identify emotions of different valences. The intonation pattern reflects subtle emotional changes that would not be detected only by intensity or duration. Studies in emotional prosody performed in different languages often include intonation to identify complex emotional states^(41-43)^. Therefore, the means and variation of f_o_ were important to differentiate emotions.

The intensity parameter is related to the amplitude of the sound wave. The highest average intensity was recorded in joy emotion and the lowest in sadness emotion. The maximum intensity recorded was in anger, then joy and the smallest in fear and sadness. Vocal intensity is one of the main parameters that guide listeners in classification^(47)^. According to the expert judges' assessment for the construction of EMOVOX-BR^(19)^, anger was the emotion with the highest percentage of success in identifying emotions and with the lowest percentage of success the fear emotion. According to the literature, anger emotion generates a greater impact on the identification of the emotions of the interlocutor, because for its production higher levels of energy are used, and it is related to changes in larynx positioning, speech speed and intensity^(8,48)^.

Previous studies have shown significant variations in parameters such as f_o_, intensity and duration, which are modulated differently according to the type of emotion expressed^(19,49)^. However, these variations can be influenced by factors such as the methodology adopted, the language spoken and even the cultural context of the participants^(19)^. The lack of standardization in the analysis protocols between studies, both in terms of equipment used, speech task used, and analysis techniques, hinders the understanding of emotional prosody in different contexts.

In this sense, it would be interesting for future studies to adopt a greater uniformity in their approaches, using emotion databases validated by specialized judges with sensitive speech and vocal measures to differentiate emotions^(50)^. In addition, the inclusion of machine learning models, which have been successfully applied in emotional prosody studies, can contribute to identify more consistent and universal patterns in acoustic characteristics related to emotions. This standardization would allow a more direct comparison between studies, as well as increasing the applicability of results in areas such as artificial intelligence and automatic emotion recognition^(49)^.

In general, the suprasegmental aspects of speech, such as temporal (duration) and dynamic characteristics (intensity and f_o_) play a crucial role in the differentiation of basic emotions. It is observed that the prosodic characteristics highlight each emotion clearly. Finally, it can be inferred that, through the analysis of acoustic-prosodic signals, it is possible to identify emotional variations in native speakers of BP. These findings broaden the understanding of emotional communication and offer new perspectives for the development of future research and technological applications, with emphasis on areas such as automatic recognition of emotions and clinical interventions. The evidence obtained reinforces the role of prosody as an essential tool in understanding emotional dynamics in human communication.

## CONCLUSION

It is possible to discriminate emotions from acoustic-prosodic measures in BP speakers. The acoustic-prosodic measures of f_o_, duration and intensity are sensitive to differentiate the various emotions.

The disgust is the one that best differed from the other emotions with higher rate of elocution, longer duration in all segments analyzed. Joy has a lower rate of utterance and higher average intensity. The fear emotion is the emotion with greater variability of f_o_, as well as presents lower values of stretches in the duration of the units. The emotion sadness is the emotion with lower values of average of f_o_, variability of f_o_ and intensity. The anger emotion presents greater energy in production, with maximum intensity recorded. The surprise is the emotion with higher average of f_o_ and with record of higher maximum frequency.
