# Supplementary material for: Acoustic-prosodic measures discriminate the emotions of Brazilian portuguese speakers
Source: Codas. 2025 Aug 4;37(4):e20240116. doi: 10.1590/2317-1782/e20240116en (PMC12323398; doi:10.1590/2317-1782/e20240116en)
Supplement: Translation

# Translation: Medidas acústico-prosódicas discriminam as emoções de falantes do português brasileiro

**DOI:** 10.1590/2317-1782/e20240116pt

**Authors:** Alexandra Christine de Aguiar, Ana Carolina Constantini, Ronei Marcos de Moraes, Anna Alice Almeida

**Affiliations:** 1 Programa de Pós-graduação em Modelos de Decisão e Saúde, Universidade Federal da Paraíba – UFPB - João Pessoa (PB), Brasil.; 2 Departamento de Desenvolvimento Humano e Reabilitação, Universidade Estadual de Campinas – UNICAMP - Campinas (SP), Brasil.; 3 Departamento de Estatística, Universidade Federal da Paraíba – UFPB - João Pessoa (PB), Brasil.; 4 Departamento de Fonoaudiologia, Universidade Federal da Paraíba – UFPB - João Pessoa (PB), Brasil.

**Keywords:** Voz, Emoção, Acústica da Fala, Prosódia, Reconhecimento da Emoção na Voz

## Abstract

**Objetivo:**

Verificar se há diferença de medidas acústico-prosódicas em diferentes estados emocionais de falantes do português brasileiro (PB).

**Métodos:**

A amostra de dados consistiu em 182 sinais de áudio produzidos por atores (profissionais ou estudantes), a partir da tarefa de fala semi-espontânea “Olha lá o avião azul” nas variadas emoções (alegria, tristeza, medo, raiva, surpresa, nojo) e emissão neutra. Foram extraídos valores das medidas acústico-prosódicas de duração, frequência fundamental e intensidade das variadas emoções. Utilizou-se o teste de comparação de Friedman para verificar se essas medidas são capazes de discriminar as emoções.

**Resultados:**

A análise acústico-prosódica revelou variações significativas entre as emoções. A emoção nojo destacou-se por apresentar a maior taxa de elocução, com valores mais altos de duração. Em contraste, a alegria exibiu uma fala mais acelerada, com menores valores de duração e maior intensidade. A tristeza e o medo foram marcados por menor intensidade e frequências mais baixas, sendo que o medo apresentou os menores valores de assimetria positiva de z-score e z-suavizado, com menor alongamento dos segmentos. A raiva se sobressaiu pela maior intensidade vocal, enquanto a surpresa registrou os valores mais altos de frequência fundamental.

**Conclusão:**

As medidas acústico-prosódicas demonstraram ser ferramentas eficazes para diferenciar emoções em falantes do PB. Esses parâmetros têm grande potencial para discernir diferentes estados emocionais, ampliam o conhecimento sobre a expressividade vocal e abrem possibilidades para tecnologias de reconhecimento de emoções, com aplicações em inteligência artificial e saúde mental.

## INTRODUÇÃO

A interface entre voz e linguagem está relacionada ao ponto de interseção entre a produção vocal e a expressão linguística. Envolve a conexão entre a capacidade de produzir sons vocais e a habilidade de utilizar esses sons de maneira estruturada e significativa, seguindo regras linguísticas específicas^(1)^. A voz é usada para transmitir a linguagem, ou seja, para expressar palavras e significados de acordo com as regras e convenções linguísticas. Ela pode diferenciar o estado emocional, expressar os traços de personalidade, sentimentos, estado de saúde física e mental, dentre outros^(2-4)^.

A linguagem influencia a forma como usamos nossa voz. A estrutura gramatical, o vocabulário e os padrões linguísticos determinam como organizamos e expressamos nossas ideias a partir da voz^(5)^. Portanto, a voz e a linguagem estão intrinsecamente relacionadas, e a sua interação é fundamental para a comunicação eficaz e expressão emocional.

Diversos estudos contribuíram para que alguns autores propusessem que seis emoções básicas (felicidade/alegria, medo, raiva, tristeza, repugnância/nojo, surpresa) fossem universalmente reconhecidas na face pelos seres humano, por apresentarem configurações específicas, expressas de forma semelhante em diferentes culturas^(6,7)^. Essas emoções quando combinadas geram um espectro de estados emocionais.

Cada estado emocional provoca modificações no trato vocal e alteram momentaneamente a fisiologia de produção da voz, que interfere no controle da respiração, no posicionamento vertical da laringe, no relaxamento relativo das pregas vocais e no posicionamento e no relaxamento dos músculos da faringe e da língua, que pode resultar na modificação da voz^(8)^. Essas variações da voz humana, quando um indivíduo vivencia um determinado estado emocional, podem envolver tanto aspectos da qualidade vocal como os atributos correlatos da prosódia^(9)^. Essa pode ser definida como um conjunto de propriedades da fala, que está além do nível do segmento, e é usualmente estudada a partir da análise de três parâmetros fonético-acústicos clássicos: duração, frequência fundamental (f_o_) e intensidade^(9-11)^. Para alguns autores, a prosódia resulta do acoplamento de uma informação sintática, semântica e discursiva e das restrições de um sistema de produção de fala^(12,13)^. As variações prosódicas transmitem informações para a significação da expressividade e marcam as características de uma dinâmica vocal, porém pouco se sabe sobre essas informações nas emoções para o português brasileiro (PB).

Alguns bancos de vozes foram desenvolvidos com a variação as emoções em diferentes línguas/culturas, como o *Berlin Database of Emotional Speech* (EMO-DB)^(14)^, *Interactive Emotional Dyadic Motion Capture* (IEMOCAP)^(15)^, *Sustained Emotionally colored Machine-human Interaction using Nonverbal Expression* (SEMAINE)^(16)^ e *Remote Collaborative and Affective interactions* (RECOLA)^(17)^. As principais características que se destacam na diferenciação das emoções nesses conjuntos de dados são as características acústicas e prosódicas, como *pitch*, energia e duração. Além disso, coeficientes cepstrais como os *Mel-Frequency Cepstral Coefficients* (MFCC) são medidas importantes para otimizar a identificação emocional^(18)^.

Recentemente foi elaborado um banco de vozes nas variadas emoções a partir de falantes nativos do PB, o EMOVOX-BR^(19)^. Houve a validação desse banco por meio do julgamento perceptivoauditivo de juízes experts. Esse estudo indicou que aspectos acústicos, como a variação do *pitch* e da *loudness*, foram essenciais para a diferenciação das emoções, além da sua valência e potência^(19)^.

A análise dos aspectos acústico-prosódicos das emoções constitui uma área de crescente interesse nas ciências da fala e da comunicação, oferecendo novas perspectivas sobre como as emoções são expressas e percebidas por meio da voz^(20-22)^, e por isso o presente estudo busca colaborar com a identificação de parâmetros vocais e fala específicos a cada estado emocional, bem como reconhecer a voz como um sinal biológico rico em informações que pode ser instrumental na detecção de padrões emocionais.

A investigação da prosódia emocional envolve tanto a forma como as modulações acústicas são produzidas quanto a maneira como são percebidas, proporcionando uma compreensão mais ampla da relação entre os processos linguísticos e emocionais na comunicação humana^(23)^, ultrapassando o conteúdo semântico das palavras. Compreender esses elementos é essencial para desvendar a complexidade da comunicação humana, na qual a dimensão emocional influencia significativamente a interação social. Além de seu impacto teórico, o estudo da prosódia emocional apresenta aplicações práticas em tecnologias avançadas de reconhecimento de emoções, inteligência artificial, e diagnósticos clínicos de transtornos da comunicação e saúde mental^(21,22)^.

Essas descobertas têm o potencial de impulsionar o desenvolvimento de sistemas de interação homem-máquina e a detecção automática de emoções pela voz, pois a prosódia envolve elementos que carregam informações importantes sobre o estado emocional do falante^(23)^. A expressão das emoções afeta esses parâmetros de forma distinta, permitindo que modelos de aprendizado de máquina utilizem essas variações para identificar e classificar emoções de maneira precisa^(24)^. Essa capacidade é essencial para criar sistemas capazes de ajustar suas respostas conforme o estado emocional do usuário, promovendo interações mais humanas e eficientes.

Esses avanços têm aplicações fundamentais para uma ampla gama de setores, como call centers, aplicativos de reconhecimento de voz, web filmes e comunicação móvel. Acredita-se que a análise de parâmetros prosódico-acústicos poderia revelar diferenças fônicas entre as variações emocionais do PB, contribuindo para o desenvolvimento de sistemas de síntese e reconhecimento de fala mais adaptados à língua e à cultura local.

Diante do exposto, surgem os questionamentos: É possível discriminar as emoções a partir de medidas acústico-prosódicas em falantes do PB? Quais aspectos prosódicos caracterizam os diferentes estados emocionais? Há diferenças na duração, frequência e intensidade entre as emoções? Assim, o objetivo desse estudo foi verificar se há diferença de medidas acústico-prosódicas em diferentes estados emocionais de falantes do PB.

## MÉTODO

Esta é uma pesquisa observacional e transversal, avaliada e aprovada pelo Comitê de Ética em Pesquisa do Centro de Ciências da Saúde de uma instituição de ensino superior do Brasil, sob número 3.304.419/2018.

O conjunto de dados para análise foi composto por 182 sinais sonoros, produzidos por 26 atores profissionais e estudantes brasileiros, de ambos os sexos, com média de idade sendo 27 anos, residentes nos Estados da Paraíba, São Paulo, Rio Grande do Sul, Ceará, Roraima, Mato Grosso e Distrito Federal, que pertencem ao Banco de Vozes Brasileiro nas Variações das Emoções - EMOVOX-BR^(19)^.

Para a construção do EMOVOX-BR^(19)^ foram gravadas amostras de voz de falantes nativos do PB expressando diferentes emoções, como alegria, tristeza, medo, raiva, surpresa, nojo e emissão neutra. Os participantes receberam instruções detalhadas sobre o procedimento de gravação e, em seguida, realizaram a coleta da voz. Foram gravadas três diferentes tarefas de fala: 1) emissão prolongada da vogal /ε/, 2) a contagem automática de 1 a 10 e 3) a fala semi-espontânea da frase “Olha lá o avião azul.”, que faz parte do Consensus Auditory Perceptual Evaluation of Voice - CAPE-V^(25)^. Cada participante executou essas tarefas nas seis emoções básicas. O que gerou o total de 1.638 sinais sonoros.

Para a validação, optou-se por utilizar a frase “Olha lá o avião azul.”, coletada via smartphone. Todos os áudios foram submetidos à análise de relação sinal-ruído (*signal to noise ratio* - SNR) e obtiveram valores superiores ao padrão de referência, ou seja, o valor obtido do SNR igual ou superior a 30dB. Foram selecionados 182 sinais de áudio para a etapa do julgamento perceptivoauditivo. Essas amostras vocais foram avaliadas por juízes fonoaudiólogos, que obtiveram altos índices de precisão na identificação das seis emoções básicas.

Este estudo analisou as medidas acústico-prosódicas dos mesmos 182 áudios utilizados na validação do EMOVOX-BR, que foi realizada a partir do julgamento perceptivoauditivo por juízes especialistas. Enquanto o estudo de validação do EMOVOX-BR enfatizou a precisão da percepção humana para identificar as emoções, o presente trabalho se aprofunda na análise quantitativa da prosódia emocional, principalmente no comportamento dos parâmetros acústicos de duração, f_o_ e intensidade. Esses parâmetros são considerados os elementos mais robustos para a discriminação de falantes e oferecem uma perspectiva técnica e mensurável dos aspectos emocionais da voz^(10,13)^.

A duração de uma emoção pode variar desde momentos breves até períodos prolongados, dependendo da intensidade do estímulo emocional, da capacidade de regulação emocional da pessoa e do contexto em que a emoção ocorre^(20)^. A duração é essencial para identificar variações na cadência da fala, estruturação do enunciado e na organização temporal de frases^(26)^.

Para análise da duração, todas as amostras de fala foram segmentadas manualmente em unidades Vogal-Vogal (unidade VV), unidades do tamanho da sílaba que compreendem um segmento que vai do início de uma vogal até o início da vogal imediatamente seguinte, incluindo as consoantes entre elas^(27)^.

A tarefa de fala utilizada foi segmentada em quatro partes, sendo: [Al] [auav] [iaNU] [az]. Para fazer a extração dessa medida, considerando o cálculo da duração normalizada das unidades VV, utilizou-se o script SG Detector^(28)^. O script apresenta uma tabela de referência com médias e desvios-padrão dos segmentos fônicos para o PB para calcular o valor da duração, z-score e o z-score suavizado das unidades VV ao longo do enunciado, o que gera uma segmentação dos grupos frasais dos enunciados. A segmentação é feita por meio do cálculo do desvio-padrão das médias de duração das unidades VV, que são normalizadas pelo cálculo de z-score.

O valor de z-score indica o número de desvios-padrão em relação à média de um ponto de informação que neste caso seriam as variações do PB, ou seja, é uma proporção do número de desvios-padrão abaixo ou acima do PB, o que significa uma pontuação bruta. Um z-score é chamado de pontuação padrão e pode muito bem ser colocado em uma curva de dispersão comum e estende-se de – 3 até + 3 desvios^(29)^.

Já os valores de z-score suavizado permitem atenuar variações locais de duração advindas da queda de duração em unidades VV pós-tônicas e/ou duração de fones muito distintos da relação de durações dos fones do PB^(27)^. Esse valor corresponde à suavização de cinco pontos aplicada à sequência de dados de z-score, o que permite observar com mais precisão as proeminências de duração. O intuito é verificar quanto os valores de duração obtidos no *corpus* do EMOVOX-BR^(19)^ variaram conforme a tabela de durações intrínsecas para os fones do PB.

Quando se considera o número de segmentos da fala unidades VV dividido pela soma total de duração destes, obtém-se a taxa de elocução^(30)^. Ela influencia a percepção do ritmo da fala, com taxas mais lentas associadas a silabação, alongamento de sons finais e pausas, enquanto taxas mais rápidas tendem a reduzir esses fenômenos^(28)^. A relação entre a duração da emissão e a taxa de elocução foi explorada para observar a variação na velocidade da fala conforme as diferentes emoções.

A f_o,_ medida em Hertz (Hz), é definida pelo número de vibrações produzidas pelas pregas vocais por segundo e está diretamente relacionada à percepção do *pitch* ^(31)^. Esse parâmetro permite uma análise detalhada da entonação e variação tonal ao longo de enunciados, fornecendo informações sobre o controle e qualidade vocal^(32)^. Para a análise da f_o_ foram medidos os valores médios, máximos, mínimos, desvio-padrão (f_o_ dp) e de variação (f_o_ range) em cada enunciado.

A intensidade, medida em decibéis (dB), está associada à força ou ao grau de ativação emocional experimentado pelo falante e está relacionada à energia vocal empregada durante a fala^(31)^. Sua mensuração ajuda a entender a energia da emissão e a modulação da força ao longo do discurso, contribuindo para estudos de prosódia e expressividade vocal^(13)^. Para a análise da intensidade foi extraído o valor médio, mínimos e máximos para cada sinal e, posteriormente, os dados foram comparados a cada emoção. As medidas de f_o_ e intensidade foram extraídas com auxílio do *plug-in* VoxMore^(33)^.

Este estudo investiga se as medidas acústico-prosódicas de duração, f_o_ e intensidade variam significativamente entre diferentes emoções, como alegria, tristeza, medo, raiva, surpresa, nojo e emissão neutra, em falantes do PB. Com base na literatura, que demonstra que as emoções influenciam os parâmetros acústicos de forma distinta^(20-22)^, o objetivo é testar essas variações utilizando um conjunto de dados de falantes nativos.

Utilizou-se o programa PRAAT, versão 5.4.04, para extração dos dados. Selecionou-se o teste de Friedman com *post hoc* de Dunn para verificar se essas diferenças nos parâmetros acústicos entre as emoções eram estatisticamente significativas, contribuindo para uma maior compreensão da relação entre os aspectos acústico-prosódicos e a expressão emocional, após os resultados extraídos e identificados os grupos que se diferenciaram, foi realizado análise descritiva das variáveis para identificar qual parâmetro prosódico e a emoção que se destacou no grupo. Todas as análises foram realizadas por meio do software *Statistical Package for Social Sciences (SPSS)*, versão 24, e utilizou-se o nível de significância de 5%.

## RESULTADOS

A análise da prosódia contemplou os dados extraídos de acordo com as unidades VV segmentadas, o contraste das variações de f_o_ e intensidade após análise.

Houve diferença significativa na comparação da duração dos segmentos de acordo com a emoção (Tabela 1). Em relação ao tempo total de duração da emissão, o maior valor foi encontrado na emoção nojo (340,63 ms), que apresentou a maior taxa de elocução. Por outro lado, a emoção alegria teve o menor valor de duração (264,73 ms), correspondendo à menor taxa de elocução, o que indica uma relação direta entre o tempo total de emissão e a velocidade da fala nas diferentes emoções.

**Tabela 1:** Comparação dos parâmetros de duração para cada unidade VV do enunciado “Olha lá o avião azul”, produzido nas variadas emoções

Duração (ms)	Emoção							p-valor
	Alegria	Medo	Tristeza	Raiva	Surpresa	Nojo	Neutra	
Média	264,73	266,81	284,83	270,32	276,7	340,63	274,83	0,024*
Al	172,92	156,08	170,73	175,04	174,81	220,88	168,5	0,001*
auav	437,73	481,81	528,46	466,27	469,04	605,15	495,69	0,066*
iaNU	212,46	204,27	215,92	205,23	198,88	239,92	206,77	0,317
az	238,81	225,08	224,19	234,73	264,08	296,58	228,35	0,000*

'Legenda: ms = milissegundos. Teste Friedman. Valores significativos (p<0,05)

Encontrou-se novamente a comprovação da maior duração em todas as quatro partes das unidades VV na emoção nojo ao analisar a duração de cada segmento individualmente (Al - 220,88 ms; auav - 605,15 ms; iaNU - 239,92 ms; az - 296,58 ms). A menor duração por segmento diferenciou significativamente todas as emoções entre si. A menor duração do primeiro segmento foi observada na emoção medo (Al - 156,08 ms), a do segundo segmento mostrou-se na alegria (auav - 437,71 ms), a do terceiro na surpresa (iaNU - 198,88 ms) e a do último segmento na tristeza (az - 224,19 ms). Não houve diferença significativa no terceiro segmento [iaNU] nas variadas emoções em todos os parâmetros relacionados à duração (Tabela 1).

Houve diferença significativa ao comparar os valores de z-score e z-suavizado do primeiro, segundo e último segmento do z-score nas variadas emoções (Tabela 2).

**Tabela 2:** Comparação dos parâmetros de z-score e z-score suavizado para cada unidade VV do enunciado “Olha lá o avião azul”, produzido nas variadas emoções

		Emoção							P-valor
		Alegria	Medo	Tristeza	Raiva	Surpresa	Nojo	Neutra	
Z-score	Média	0,95	0,74	1,23	0,98	1,10	2,74	0,99	0,014*
	Al	3,37	2,52	3,26	3,48	3,47	5,79	3,15	0,024*
	auav	0,22	0,85	1,54	0,62	0,98	2,67	1,06	0,079*
	iaNU	-0,63	-0,99	-0,48	-0,95	-1,22	0,56	-0,88	0,317
	az	0,83	0,55	0,56	0,75	1,19	1,95	0,63	0,001*
Z-score suavizado	Média	0,94	0,78	1,28	1,00	1,13	2,8	1,03	0,015*
	Al	2,32	1,97	2,69	2,53	2,64	4,75	2,45	0,002*
	auav	1,27	1,41	2,11	1,57	1,81	3,71	1,75	0,028*
	iaNU	-0,17	-0,31	0,13	-0,29	-0,3	1,26	-0,19	0,174
	az	0,34	0,05	0,20	0,19	0,39	1,49	0,13	0,055*

Legenda: Teste Friedman; *Valores significativos (p<0,05)

A Tabela 2 apresenta a emoção nojo com os maiores valores de assimetria positiva de z-score e z-suavizado. Verificou-se que a emoção medo tem os menores valores de assimetria positiva entre todas as emoções. É possível observar valores negativos de z-score e z-suavizado no terceiro segmento [iaNU] nas emoções alegria, medo, tristeza, raiva surpresa e emissão neutra. Já na emoção nojo ocorreu um alongamento dos segmentos comparados aos valores de referência (Tabela 2).

Observou-se significância na comparação de todas as medidas acústicas relativas à frequência da voz nas variadas emoções analisadas. Verificou-se que a maior média de f_o_ foi na emoção surpresa (284,43 Hz), seguida da alegria (268,54 Hz), e a menor na tristeza (160,91 Hz) e neutra (163,44 Hz). A maior frequência máxima foi apresentada na emoção surpresa (360,36 Hz) e a menor frequência mínima foi marcada pela tristeza (77,91 Hz). A emoção medo obteve o maior valor de f_o_ range (178,11 Hz) e a menor na emoção tristeza (116,73 Hz) (Tabela 3).

**Tabela 3:** Comparação dos valores de f_o_ para as variadas emoções (média, mínimo, máximo e range)

f_o_ (Hz)	Emoção							P-valor
	Alegria	Medo	Tristeza	Raiva	Surpresa	Nojo	Neutra	
Média	268,54	228,29	160,91	213,23	284,43	188,47	163,44	0,000*
mín	166,42	102,05	77,91	137,10	183,61	135,15	80,91	0,000*
máx	344,10	281,86	203,80	287,79	360,36	269,94	194,65	0,000*
range	174,67	178,11	116,73	145,79	176,75	134,79	122,88	0,000*

Legenda: Teste Friedman; *Valores significativos (p<0,05); fo = frequência fundamental; Hz = hertz

Houve comparações das amostras independentes da intensidade média, mínima e máxima significativas nas variadas emoções (Tabela 4). A maior intensidade máxima registrada foi na raiva (82,35 dB), seguida da alegria (66,79 dB), e a menor no medo (35,5 dB) e tristeza (36,78 dB). Verificou-se diferença estatisticamente significativa na comparação das médias da intensidade, com a intensidade mais alta registrada na emoção de alegria (66,79 dB) e a mais baixa na emoção de tristeza (60,23 dB).

**Tabela 4:** Comparação dos valores de intensidade para as variadas emoções (média, mínimo e máximo)

Intensidade (dB)	Emoção							P-valor
	Alegria	Medo	Tristeza	Raiva	Surpresa	Nojo	Neutra	
Média	66,79	63,8	60,23	65,58	65,08	61,91	61,28	0,001*
mín	55,29	35,5	36,78	54,87	47,86	43,12	41,89	0,001*
máx	80,11	79,64	77,02	82,35	79,72	77,77	79,8	0,001*

Legenda: Teste Friedman; *Valores significativos (p<0,05); dB = decibel

O Quadro 1 exibe uma síntese das variações prosódicas presentes nas diversas emoções, dentro do contexto de falantes do PB, ou seja, as características que mais marcam cada emoção e as diferencia das demais

**Quadro 1:** Variações prosódicas nas diferentes emoções do português brasileiro

Emoções	Medidas acústico-prosódicas		
	Duração	Frequência	Intensidade
Alegria	↓	↑	↑
Raiva	ND	ND	↑
Surpresa	ND	↑	ND
Tristeza	ND	↓	↓
Medo	↓	ND	↓
Nojo	↑	ND	ND
Neutra	ND	↓	ND

**Legenda**: ↑ = maior; ↓ = menor; ND = não discrimina

## DISCUSSÃO

As características dos parâmetros acústico-prosódicos apresentam variações que repercutem na expressão das emoções. O conhecimento dessas medidas contribui para construção da definição sobre as variações emocionais comuns nos sinais vocais, podem auxiliar nos aspectos de diagnóstico clínico e favorecem a criação de modelos de reconhecimento das emoções a partir da voz^(34)^.

Foram desenvolvidos diversos bancos de vozes que incorporam a variação emocional, que abrangem populações de atores em diferentes idiomas e culturas^(14-17)^. No entanto, esses bancos de vozes geralmente se concentram apenas na análise das variações acústicas tradicionais e pouco se tem sobre outros tipos de medidas, como: aprofundamento nas medidas acústico-prosódicas e perceptuais por parte de fonoaudiólogos e no impacto do julgamento por ouvintes especialistas em voz, além da possibilidade de encontrar reconhecimento de padrões de voz comuns a cada uma das emoções.

A inclusão de medidas prosódicas na análise da variação emocional da voz, em um conjunto de dados validado por juízes experts é fundamental para uma análise abrangente e precisa da expressão emocional humana^(35)^. Isso aumenta a validade dos dados e melhora a capacidade de generalização para diferentes contextos e populações, além de aprimorar a eficácia e a robustez dos modelos de reconhecimento automático de emoções. Assim, pode contribuir para uma compreensão mais holística dos processos emocionais humanos e de como eles são expressos e percebidos através da fala^(36)^.

A prosódia tem sua dimensão pautada nos aspectos suprassegmentais da fala, que se relacionam às variações de duração, frequência e intensidade^(10,37)^. O presente estudo buscou explorar se há diferenças das variações prosódicas em diferentes emoções a partir da voz, os dados relatados nesse estudo confirmaram que esses parâmetros são fatores importantes para definir as emoções por meio da análise objetiva dos sinais vocais. Dos dezenove parâmetros que apresentaram resultados significativos, doze dizem respeito à estrutura rítmica das variedades estudadas, quatro referem-se à f_o_ e três à intensidade, o que mostra que os três parâmetros clássicos de estudo da prosódia apresentam distinções entre as variadas emoções.

Houve diferença entre as medidas de duração na tarefa de fala nas variadas emoções. O nojo apresentou maiores valores na média da duração, bem como em todas as segmentações das unidades VV, ou seja, foi a emoção com maior taxa de elocução de fala. Quanto à menor média de duração, a emoção alegria apresentou menores valores, sendo assim é a emoção com velocidade de fala mais acelerada. Mudanças na velocidade de fala alteram as características fonéticas do sinal, dessa forma torna-se um parâmetro capaz de diferenciar as emoções^(38)^.

Ao analisar o parâmetro de menor duração por segmento, observou-se que cada segmento apresentou menor duração em emoções diferentes, variou entre medo, alegria, surpresa e tristeza. Nem sempre os falantes de uma língua expressam emoções da mesma forma, com os mesmos níveis de ativação^(39)^. Cada estado emocional pode ser definido como uma combinação linear de alguns eixos, como os de ativação (ou excitação) e potência (ou poder). Ativação mede o grau de excitação do indivíduo em expressar a emoção. Potência diz respeito à força da emoção^(40)^. Então, cada uma das emoções básicas pode gerar diferentes níveis de ativação e potência no falante, a depender da sua manifestação pode provocar diferenciações na velocidade de fala.

Para os dados obtidos de z-score e z-suavizado, observou-se diferenças significativas em relação às variações de fala do PB entre as emoções. A emoção nojo apresentou maiores valores de assimetria positiva. Esse fato indica que os falantes aumentam mais a duração de algumas unidades VV durante a emissão dessa emoção. Significa que no nojo houve mais alongamentos na duração das unidades, o que resulta na assimetria de valor positivo mais elevada, e assim, relaciona-se a uma fala mais lentificada^(27)^. Já o medo foi a emoção com menores valores de assimetria positiva entre todas as emoções, isto é, há menos alongamentos na duração das unidades.

Quanto aos valores negativos de z-score e z-suavizado encontrados no terceiro segmento [iaNU] nas emoções alegria, medo, tristeza, raiva, surpresa e emissão neutra. Esses valores abaixo dos padrões de referência significam que houve um encurtamento na duração dos segmentos para essas emoções^(27)^. A aceleração do segmento [iaNU] foi uma estratégia utilizada na emissão das emoções, portanto não apresentou diferença significativa na comparação. No caso da emoção nojo, ocorreu um alongamento dos segmentos comparados aos valores de referência, confirmando assim os resultados encontrados para a taxa de elocução.

Estudos desenvolvidos em diferentes línguas revelam que a elocução mais rápida, está associada a emoções de maior excitação. Por outro lado, uma fala mais lenta é geralmente vinculada a estados de reflexão ou calma. Em línguas tonais, como o mandarim, a taxa de elocução não só afeta a prosódia, mas também pode alterar o significado das palavras, sendo crucial para a interpretação correta das intenções do falante^(41)^. As variações culturais e regionais desempenham um papel importante na modulação da velocidade da fala, além de indicar emoções, a taxa de elocução pode refletir normas culturais e linguísticas específicas^(41-43)^. Portanto, a análise da duração oferece uma compreensão mais profunda dos padrões emocionais e comunicativos, variando de acordo com o idioma e o contexto cultural.

Verifica-se que a média e os valores máximos de f_o_ permitem distinguir a emoção surpresa e a alegria, o que as posiciona entre as faixas superiores de f_o_. Essas emoções são caracterizadas por uma maior variação tonal, típica de estados emocionais de maior excitação ou positivos^(44)^, ao contrário da tristeza e neutra, que apresentam valores mínimos e média de f_o_ baixo. A alegria é uma emoção de valência positiva e a surpresa é uma emoção bivalente. As características de f_o_ discriminam emoções na dimensão de valência com maior precisão^(45)^. Para o EMOVOX-BR^(19)^, os juízes experts indicaram que a surpresa desse banco era uma emoção de valência positiva. Esse fato justifica, pois, as emoções de valência positiva têm como característica variação do *pitch* em curva ascendente. A f_o_ está diretamente relacionada à função laríngea, portanto, alterações de entonação e fluxo de ar produzida pelo trato vocal devido ao estado emocional podem ser identificadas na análise acústico-prosódica do sinal vocal^(46)^. A variação de f_o_ é importante no medo, surpresa e alegria, pois a afastam da tristeza, nojo e neutra.

A variação no *pitch* ao longo da fala mostrou-se essencial para identificar emoções de diferentes valências. O padrão entonacional reflete mudanças emocionais sutis que não seriam detectadas apenas pela intensidade ou duração. Estudos em prosódia emocional realizados em diferentes línguas, frequentemente incluem a entonação para identificar estados emocionais complexos^(41-43)^. Portanto, as médias e a variação de f_o_ foram importantes para diferenciar as emoções.

O parâmetro de intensidade está relacionado à amplitude da onda sonora. A maior média de intensidade foi registrada na emoção alegria e a menor na emoção de tristeza. A intensidade máxima registrada foi na raiva, em seguida alegria e a menor no medo e tristeza. A intensidade vocal é um dos principais parâmetros que guiam os ouvintes na classificação^(47)^. Segundo a avaliação dos juízes especialistas para construção do EMOVOX-BR^(19)^, a raiva foi a emoção com maior percentual de acerto na identificação das emoções e com menor percentual de acerto a emoção medo. Segundo a literatura, a emoção raiva gera um impacto maior na identificação das emoções do interlocutor, pois para sua produção é empregado níveis mais elevados de energia, e está relacionada a alterações de posicionamento de laringe, velocidade de fala e intensidade^(8,48)^.

Estudos prévios mostraram variações significativas em parâmetros como f_o_, intensidade e duração, que são modulados de maneira diferente conforme o tipo de emoção expressa^(19,49)^. No entanto, essas variações podem ser influenciadas por fatores como a metodologia adotada, a língua falada e até mesmo o contexto cultural dos participantes^(19)^. A falta de padronização nos protocolos de análise entre os estudos, tanto em termos de equipamentos utilizados, tarefa de fala utilizada, quanto nas técnicas de análise, dificulta a compreensão da prosódia emocional em diferentes contextos.

Nesse sentido, seria interessante que futuros estudos adotassem uma maior uniformidade em suas abordagens, utilizando bancos de dados de emoções validados por juízes especializados com medidas vocais e de fala sensíveis para diferenciar as emoções^(50)^. Além disso, a inclusão de modelos de aprendizado de máquina, que vêm sendo aplicados com sucesso em estudos de prosódia emocional, pode contribuir para identificar padrões mais consistentes e universais nas características acústicas ligadas às emoções. Essa padronização permitiria uma comparação mais direta entre os estudos, além de aumentar a aplicabilidade dos resultados em áreas como a inteligência artificial e o reconhecimento automático de emoções^(49)^.

De modo geral, os aspectos suprassegmentais da fala, como características temporais (duração) e dinâmicas (intensidade e f_o_) desempenham um papel crucial na diferenciação das emoções básicas. Observa-se que as características prosódicas destacam cada emoção de forma clara. Por fim, pode-se inferir que, por meio da análise de sinais acústico-prosódicos, é possível identificar variações emocionais em falantes nativos do PB. Esses achados ampliam o entendimento sobre a comunicação emocional e oferecem novas perspectivas para o desenvolvimento de futuras pesquisas e aplicações tecnológicas, com destaque para áreas como reconhecimento automático de emoções e intervenções clínicas. As evidências obtidas reforçam o papel da prosódia como uma ferramenta essencial na compreensão das dinâmicas emocionais na comunicação humana.

## CONCLUSÃO

É possível discriminar as emoções a partir de medidas acústico-prosódicas em falantes do PB. As medidas acústico-prosódicas de f_o_, de duração e de intensidade são sensíveis para diferenciar as variadas emoções.

O nojo é o que melhor se diferenciou das demais emoções com maior taxa de elocução, maior duração em todos os segmentos analisados. A alegria apresenta menor taxa de elocução e de maior média de intensidade. A emoção medo é a emoção com maior variabilidade de f_o_, bem como apresenta menores valores de alongamentos na duração das unidades. A emoção tristeza é a emoção com menores valores de média de f_o_, variabilidade de f_o_ e de intensidade. A emoção raiva apresenta maior energia na produção, com máxima intensidade registrada. A surpresa é a emoção com maior média de f_o_ e com registro de maior frequência máxima.
